# Discovery, Significance, and Utility of JAK2 Mutation in Squamous Cell Carcinoma of the Lung

**DOI:** 10.7759/cureus.25913

**Published:** 2022-06-13

**Authors:** Jasmin Hundal, Nerea Lopetegui-Lia, James Vredenburgh

**Affiliations:** 1 Internal Medicine, University of Connecticut, Farmington, USA; 2 Hematology and Oncology, Cleveland Clinic, Cleveland, USA; 3 Hematology and Oncology, St. Francis Hospital and Medical Center, Hartford, USA

**Keywords:** genetic mutation, myeloproliferative neoplasms, liquid biopsies, non small cell lung cancer, jak2 mutation

## Abstract

Lung cancer continues to be the leading cause of cancer-related deaths worldwide. Many studies show lung cancer is a histologically and molecularly heterogeneous group, even within the same histological subtype. *Liquid biopsies* are a new tool that can identify targetable genomic mutations and impact management. JAK2 p.V617F is a mutation commonly found in myeloproliferative neoplasms but rarely identified in non-small cell lung cancer (NSCLCs). The significance of Janus Kinase (JAK2) mutation in lung cancer is not clearly understood. However, it is thought that it may have a role in treating solid tumors, such as lung cancer. We present two cases of patients diagnosed with NSCLC who were discovered to have JAK2 V617F mutation on liquid biopsy.

## Introduction

Lung cancer accounts for roughly 58% of the total cancer deaths worldwide. It is a histologically and molecularly heterogeneous group for which diagnostic tools have been developed to improve associated morbidity and mortality [1,2]. Liquid biopsies, also known as circulating free DNA (cfDNA), are a new tool that can identify targetable genomic mutations, impact management, and overall survival [1,3,4]. Treatment of different subtypes of lung cancer is a rapidly evolving field, as we now rely on identifying molecular alterations and precise somatic mutations that lead to tumor growth. This has been possible thanks to next-generation sequencing (NGS) technologies which have helped individualize cancer treatment [5,6].

The majority of the newly diagnosed lung cancers are non-small cell lung cancer (NSCLC), of which 30% are squamous cell carcinoma (SCC) and 40% are adenocarcinoma [6]. NSCLC has the most evidence for circulating tumor DNA (ctDNA) [7-8]. The Non-invasive vs. Invasive Lung Evaluation (NILE) study reported that adding plasma ctDNA NGS increased the positive identification of a guideline-recommended genomic biomarker by 48% [7].

In contrast to adenocarcinoma of the lung, where targeted mutations have been described extensively with reported high rates of somatic mutations and about 18 statistically significant genetic mutations, it is only recently that new genomic abnormalities are being discovered in SCC, with 11 statistically substantial genetic mutations [2]. 

JAK is a cytoplasmic non-receptor tyrosine kinase and is part of the JAK/STAT pathway required for cell survival and differentiation [9]. JAK2 p.V617F is a mutation commonly found in myeloproliferative neoplasms (MPNs) but rarely identified in NSCLCs [5,9]. The JAK2 p.V617F mutation activates JAK2 kinase and, therefore, the JAK kinase - signal transducer and activator of the transcription signaling (JAK/STAT) pathway. The JAK/STAT pathway has been increasingly recognized in lung cancer, regulating multiple cellular pathways, and it is also essential in hematological and solid cancer genesis and development. Persistent activation of Stat3 is known to be oncogenic and vital for the survival of some cancer cells. Stat3 promotes the overexpression of genes that encode anti-apoptotic proteins, cell-cycle regulators, and angiogenic factors. Its activation occurs via JAK kinases. Therefore, inhibition of JAK2 activity leads to termination of Stat3 nuclear translocation and tumorigenesis [10].

The significance of Janus Kinase JAK2 mutation in lung cancer is not clearly understood. A study by Xu et al. indicates that the dysregulation, mutation, and amplification of JAK2 are associated with cancer progression [9]. We present two cases of patients diagnosed with NSCLC who were discovered to have JAK2 V617F mutation on liquid biopsy.

## Case presentation

Case 1

**Figure 1:**
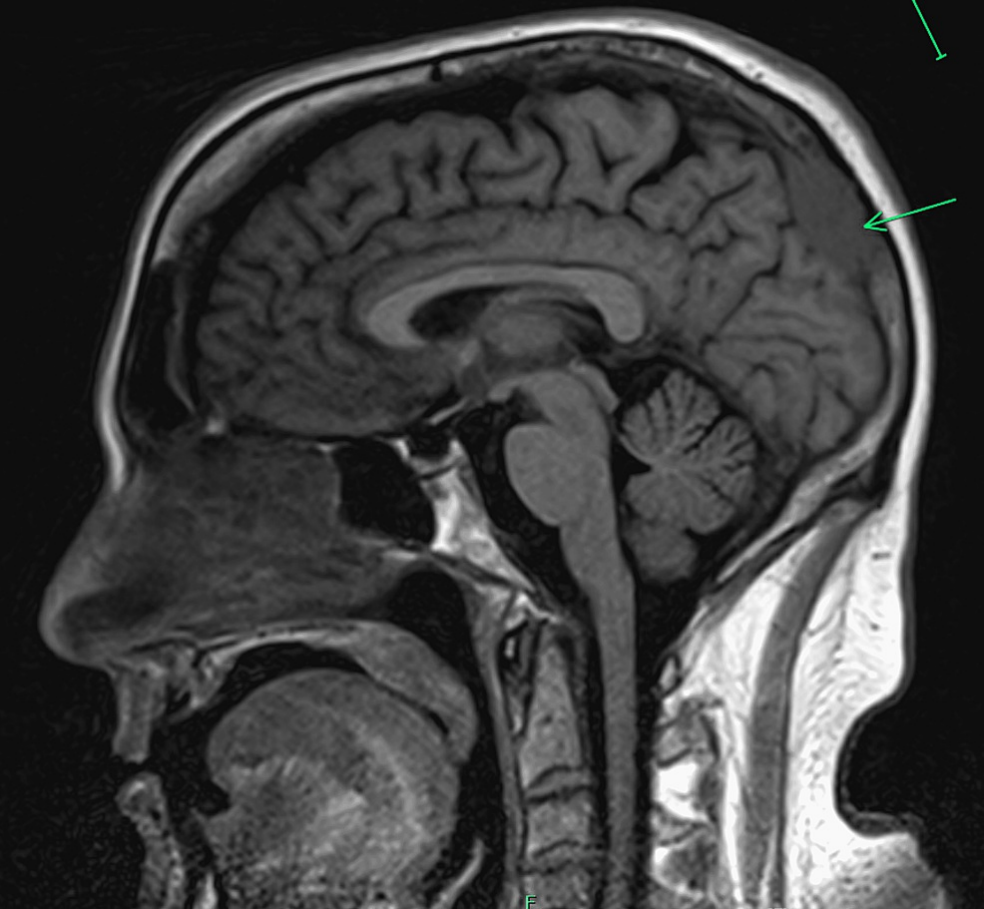
Magnetic Resonance Imaging (MRI) of the brain demonstrating bony metastatic deposit to the superior occipital bone measuring up to 3.2 x 1.6 x 2.1 cm with involvement of the dura. No vasogenic edema within the underlying brain parenchyma in the region of the bony metastatic deposit.

A 58-year-old man, a former cigarette smoker of 30 pack-year, who had quit 25 years prior, developed a severe headache that persisted for four weeks. He underwent a head computed tomography (CT) study, which showed a calvarial mass with impingement of the dura. Brain magnetic resonance imaging (MRI) revealed a 3.2 x 2.1 x 1.5 cm lesion involving the occipital bone (Figure 1).

**Figure 2:**
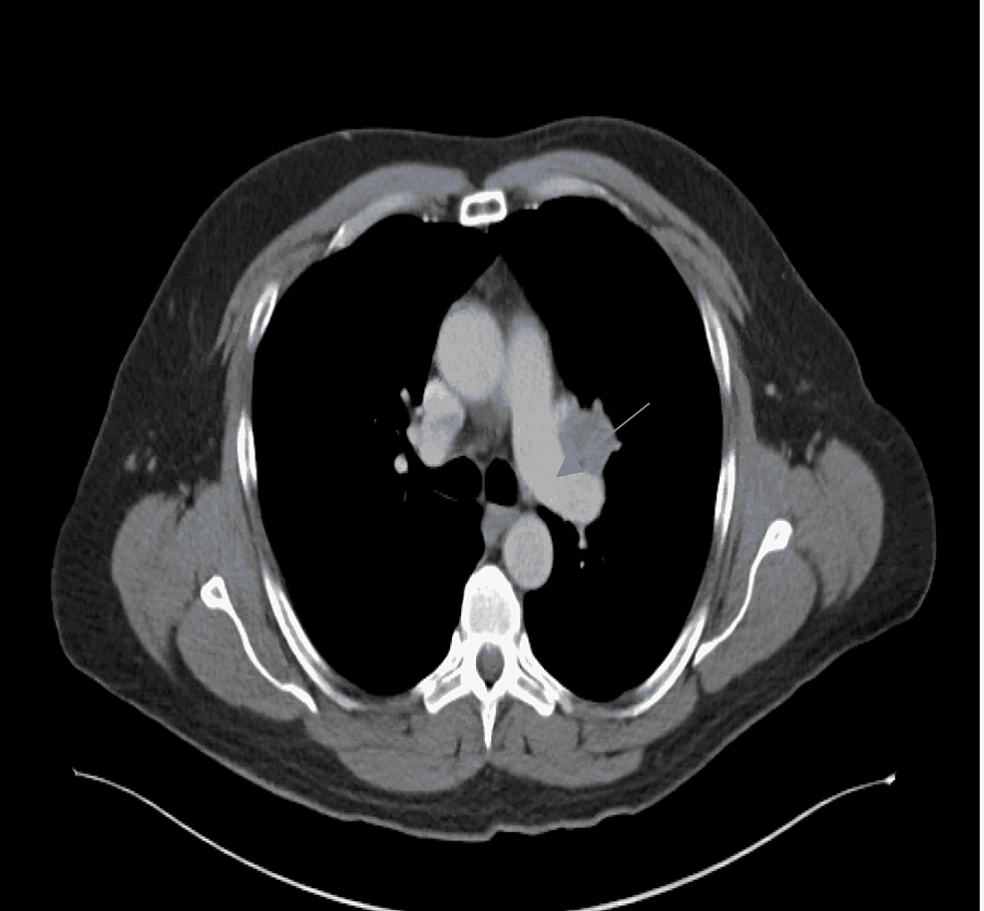
Computed Tomography (CT) of the chest demonstrating a 2.2 cm x 2.8 cm left perihilar stellate-shaped mass lesions with a probable invasion of adjacent lingular bronchus and possibly left main pulmonary artery.

He proceeded with a chest and abdomen CT where a 2.8 x 2.2 cm left perihilar stellate mass abutting the pulmonary artery was observed (Figure 2).

**Figure 3:**
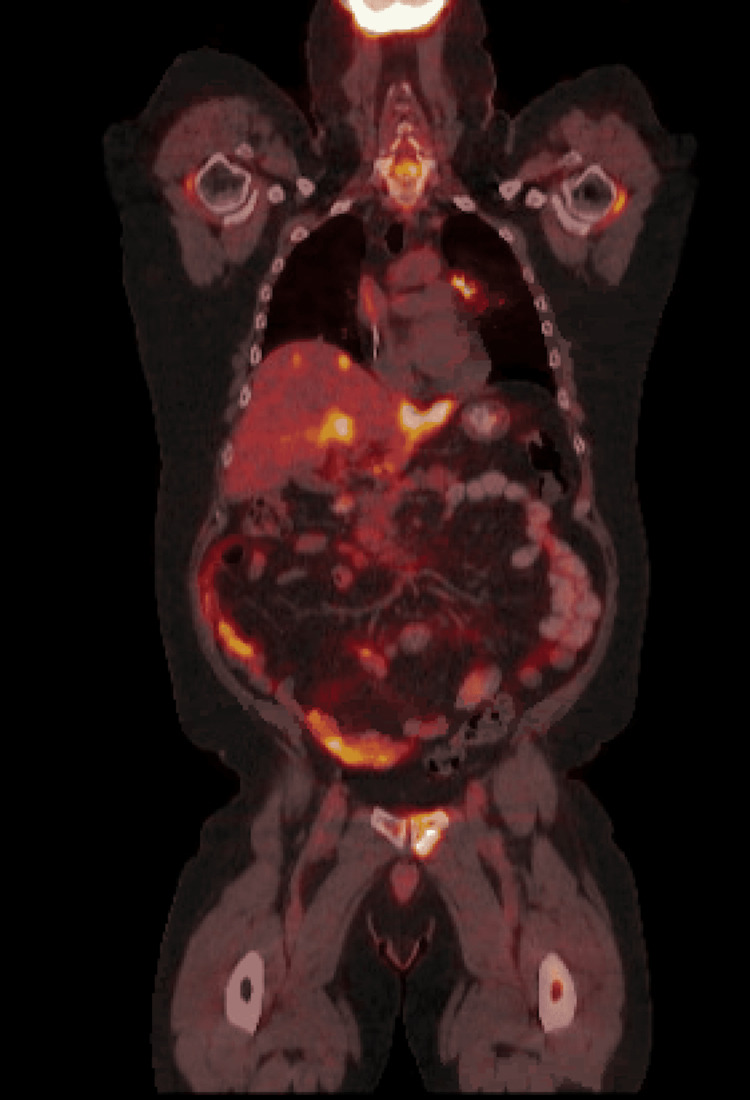
Positron emission tomography demonstrating widespread metastasis, including left and right lobe of the liver and multiple bone metastases.

The pathology revealed a moderate-to-poorly differentiated squamous cell carcinoma (SCC) of the lung, and a biopsy of the skull lesion confirmed it was a metastatic lesion related to lung SCC. He underwent radiation therapy (RT) (30 Gy in 10 fractions) to the skull metastasis but had tumor progression. He then received 30 Gy to the whole brain. Follow-up brain MRI seven months after completion of RT revealed progression of dural and calvarial metastases. In addition, he received 60Gy to the left perihilar tumor and mediastinal disease with weekly carboplatin and docetaxel for six total cycles. He was treated like a stage IIIB NSCLC given the oligo-metastatic condition. The patient continued to have confusion and changes in his mental status. There was a concern for carcinomatous meningitis, but the cytology from lumbar puncture was negative on two occasions. Follow-up CT of the chest, abdomen, and pelvis and subsequent PET scan confirmed diffuse progression of the disease (Figure 3).

Given his progression, the patient was started on carboplatin and gemcitabine. A liquid biopsy revealed 1.8% cell-free DNA (cfDNA) of JAK2 V617F and 0.4% TP53 G266V without detecting high microsatellite status instability. Despite the detection of JAK2 V617F mutation, his insurance company denied ruxolitinib, which is considered an experimental treatment in this case. The patient's disease rapidly progressed, and he clinically deteriorated. At that time, he was transitioned to hospice care and was deceased shortly after that.

Case 2

A 71-year-old man with a prior history of opiate use disorder on methadone presented to the emergency department for progressive weakness in bilateral lower extremities and numbness from his chest down. He was found to have right pulmonary neoplasm with invasion and destruction of the adjacent thoracic spine with no distant metastasis. Biopsy of the spinal lesion showed adenocarcinoma of the lung. He was diagnosed with Stage IIIC, T4 N0 M0, adenocarcinoma of the right upper lobe of the lung with direct invasion in T3-T6 resulting in cord compression. A liquid biopsy was obtained, which detected JAK2 V617F 2.3%, TP53 V197L 4.8%, MPL W515L 0.6% cfDNA, and high KRAS amplification. He underwent a T1-T8 fusion, T4-T5 corpectomy, and T6 kyphoplasty followed by radiation therapy, weekly carboplatin, and docetaxel. Despite the 6 to 7 cycles plan, he only received two weekly doses of the radiosensitizing chemotherapy because he became septic with Klebsiella pneumoniae bacteremia. After he recovered, he completed radiation therapy, but his functional status declined. After a discussion with the patient, it was decided not to continue chemotherapy. The patient had bilateral lower extremity weakness with motor strength 1/5 on the left lower extremity. He remained in a wheelchair and bedbound because of this. A few months later, he was admitted to the hospital with a large, stage IV, infected sacral decubitus ulcer with associated fasciitis and myositis. At that time, he was deemed not a candidate for further therapy for his lung cancer.

## Discussion

A tissue biopsy can be difficult and risky to obtain in many instances; therefore, cfDNA, acquired and analyzed from the peripheral blood, can be a convenient way to generate a comprehensive mutational profile. Several studies have assessed the feasibility, accuracy, and reproducibility of quantifying mutations from plasma samples with results comparable to those obtained with the standard of care tissue genotyping [11-12]

Obtaining liquid biopsies in lung cancer is relevant, as treatment is greatly determined by tumor biology and its targetable genomic mutations [13]. Liquid biopsies have other applications that include but are not limited to treatment response monitoring, recurrence detection, identification of resistance mechanisms, genetic changes that could lead to treatment resistance, and outcome prognostication [3]. A significant concordance was observed between the amount of cfDNA, primary myelofibrosis (PMF), leukocyte count, MPL-mutated groups, and thrombotic events at diagnosis and follow-up [3].

At the somatic level, cfDNA studies detect tumors, identify drug targets, determine molecular heterogeneity, and monitor tumor dynamics and response by non-invasive methods [14-15]. Although cfDNA was not intended to recognize germline mutations, new studies show that they can be accurately identified in 1-13% of cases [14-15]. Due to the sampling methodology, it is difficult to determine quantitatively whether cfDNA represents somatic vs. germline mutations and should not replace validated genetic cancer gene testing [14]. Still, it can be an additional helpful tool for patient care and, at times, the only way to evaluate the molecular profile which can impact treatment. 

In Slavin et al. and Stout et al., all variants suspected of germline origin had an allele fraction of 40% to 60%. This cut-off was selected based on the expected 50% mutant allele fractions (MAFs) with a standard deviation of 2.3% for germline heterozygous alterations [14-15]. This is because circulating tumor cfDNA sequencing is from mostly leukocyte-derived DNA. Circulating DNA NGS for somatic tumor mutations may identify heterozygous germline mutations at approximately 50% MAF [14-15]. Somatic alterations related to ctDNA or clonal hematopoiesis typically occur at lower MAFs (usually allele fraction < 40%) and, therefore, are generally distinguishable from germline mutations [14-15]. As in our patient, the percentage of cfDNA was significantly below 50%, indicating a higher likelihood of being a somatic mutation. 

Gain or loss of function of JAK2 has been associated with a significant increase or reduction of programmed death ligand-1 (PD-L1) expression, which ultimately can affect response to immunotherapy. Some studies suggest that JAK2 p.V617F confers sensitivity to JAK inhibitors and anti-PD1 immunotherapy by increasing PD-L1 expression [16]. JAK2 gain of function mutation is associated with significantly elevated PD-L1 expression [17]. JAK2 p.V617F was identified in our first patient's liquid biopsy (Guardant360TM), with 0.4% TP53 G266V; and JAK2 V617F 2.3%, TP53 V197L 4.8%, MPL W515L 0.6% of DNA and high KRAS amplification in our second patient.

JAK2 p.V617F is an activating mutation commonly found in MPNs such as polycythemia vera (PV) (>90% of cases), essential thrombocythemia (ET) (60% of cases), and PMF [18]. The role of the JAK/STAT pathway and JAK inhibitors are being investigated in clinical trials. The reported prevalence of JAK2 mutation in NSCLC is rare. Still, activity is noted to be upregulated in epidermal growth factor receptor (EGFR) tyrosine kinase inhibitor (TKI) resistant, EGFR mutant NSCLC cells, and JAK2 inhibition [3,5,19-20]. Xu et al. demonstrated that upregulation of JAK2 is associated with lung adenocarcinoma lymph node metastasis, and downregulation was associated with suppression of proliferation, migration, and invasion of the lung adenocarcinoma cells [9]. 

Another study by Liu et al. revealed that p-JAK1 expression in early-stage NSCLC conferred a poor prognosis. However, there was no significant difference in survival time in the late stages [21]. The study indicated that JAK2 expression is an independent predictor of poor prognosis and significantly affects the survival time in early-stage NSCLC [21]. This has been further affirmed by Hu et al., in which 94 resected lung cancer samples were evaluated, and those with PD-L1 and JAK2 gene mutation had a worse prognosis [22].

There is an ongoing phase I trial with JAK2 inhibitor AZD1480 to assess its safety and tolerability, as well as further evaluation of its pharmacokinetics in patients with advanced solid malignancies in the escalation phase and EGFR or reactive oxygen species (ROS) mutant NSCLC or non-smokers with lung metastasis in the expansion phase [5,23].

NSCLC patients with EGFR mutations are treated with EGFR-TKI such as erlotinib. However, resistance to erlotinib has been seen in patients with secondary somatic EGFR mutations. A study by Zhang et al. evaluated the effect of a JAK2 inhibitor on the tumor growth of erlotinib-resistant NSCLC cells. Results indicated that the JAK2 inhibitor significantly increased the cytotoxic effect of erlotinib on erlotinib-resistant NSCLC cells [24]. It also stimulated erlotinib-induced apoptosis, downregulated EGFR expression, and inhibited tumor growth of erlotinib-resistant NSCLC cells. This study concluded that JAK 2 inhibitors might be a possible adjuvant agent for NSCLC patients during erlotinib treatment [5,24].

Researchers have also demonstrated a strong association between Stat3 activation and survivin expression in cancer cells. Survivin is a member of the inhibitor of an apoptosis protein family, and it prevents apoptosis but also appears to mediate resistance to radiation therapy. Sun et al. studied TG101209, a small-molecule inhibitor of JAK2, which was demonstrated to affect survivin expression in lung cancer and possibly act as a radiosensitizing agent [25]. 

Another study reported that cisplatin-resistant cells with higher JAK2 and Stat3 expression combined with cisplatin and ruxolitinib suppressed cell growth and downregulated caspase-3 expression [19]. Caspase-3 is an enzyme with a critical function in apoptosis, as it enables DNA fragmentation and cytoskeletal protein degradation [26]. 

Despite not having a clear understanding of the significance of JAK2 mutation in lung cancer, some hypotheses indicate that activating JAK2 p.V617F mutation could increase sensitivity to both JAK inhibitors and anti-PD1 immunotherapy [11] and hence, have a role in the treatment of some solid tumors. The study by Guibert et al. describes how the response to immune checkpoint inhibitors can be predicted using companion biomarkers [6].

Is it often difficult to determine the significance of ctDNA results, especially when they are uncommon and unforeseen? False-positive results can occur due to sequencing errors, artifacts, germline variants, or the presence of somatic mutations in hematopoietic stem cells because of clonal hematopoiesis [7-8]. However, identification of mutations via cfDNA should be factored in when considering treatment options. These findings have shown not to be incidental [27]. They are essential in cases where patients have already been treated with all approved treatment options, and despite that, their malignancy is progressing, and they are experiencing clinical deterioration. Essential elements of molecular pathways and their function in oncogenesis are summarized in Table 1.

**Table 1:** Vital elements of molecular pathway and its function in oncogenesis

Molecular pathway	Function
JAK-STAT pathway	Regulates multiple cellular processes Important cancer genesis and development
STAT3	Promotes the overexpression of genes that encode anti-apoptotic proteins, cell-cycle regulators, and angiogenic factors associated with surviving expression. Survivin prevents apoptosis and mediates resistance to radiation therapy
JAK2 expression	It is an independent predictor of poor prognosis Gain or loss of function of JAK2 has been associated with a significant increase or reduction of PD-L1 expression

## Conclusions

There are many ongoing efforts to identify genetic markers that will respond to immunotherapy, targeted therapy, or other new treatment options. These cases highlight the importance of obtaining a liquid biopsy and a complete genetic sequence profiling to identify specific driver mutations that could lead to better treatment outcomes. Furthermore, as we discover the significance of JAK2 mutation in NSCLC, this may lead to a different or additional management approach and outcome shortly.
